# *NF1* mutations identify molecular and clinical subtypes of lung adenocarcinomas

**DOI:** 10.1002/cam4.2175

**Published:** 2019-06-14

**Authors:** Camille Tlemsani, Nicolas Pécuchet, Aurelia Gruber, Ingrid Laurendeau, Claire Danel, Marc Riquet, Françoise Le Pimpec‐Barthes, Elizabeth Fabre, Audrey Mansuet‐Lupo, Diane Damotte, Marco Alifano, Armelle Luscan, Benoit Rousseau, Dominique Vidaud, Jennifer Varin, Beatrice Parfait, Ivan Bieche, Karen Leroy, Pierre Laurent‐Puig, Benoit Terris, Helene Blons, Michel Vidaud, Eric Pasmant

**Affiliations:** ^1^ Service de Génétique et Biologie Moléculaires, Hôpital Cochin, Hôpitaux Universitaires Paris Centre Assistance Publique‐Hôpitaux de Paris (AP‐HP) Paris France; ^2^ EA7331, Faculté de Pharmacie de Paris Université Paris Descartes Paris France; ^3^ INSERM UMR‐S1147 Université Sorbonne‐Paris‐Cité Paris France; ^4^ Service d'Anatomopathologie Hôpital Bichat, AP‐HP Paris France; ^5^ Service de Chirurgie Thoracique Hôpital Européen Georges Pompidou (HEGP), AP‐HP Paris France; ^6^ Service d'Oncologie Médicale Hôpital Européen Georges‐Pompidou (HEGP), AP‐HP Paris France; ^7^ Service d'Anatomopathologie Hôpital Cochin, Hôpitaux Universitaires Paris Centre, AP‐HP Paris France; ^8^ Service de Chirurgie Thoracique Hôpital Cochin, Hôpitaux Universitaires Paris Centre, AP‐HP Paris France; ^9^ Service d'Oncologie Médicale hôpital Henri‐Mondor, AP‐HP Créteil France; ^10^ Faculté de médecine de Créteil Université Paris Est Créteil France; ^11^ Faculté de médecine de Créteil Institut Mondor de recherche biomédicale, Inserm U955 équipe 18 Créteil France; ^12^ Service de Génétique Institut Curie Paris France; ^13^ Service de Biochimie, Pharmacologie et Biologie Moléculaire Hôpital Européen Georges‐Pompidou (HEGP), AP‐HP Paris France

**Keywords:** lung adenocarcinoma, molecular subtype, next generation sequencing, *NF1*, RAS‐MAPK pathway

## Abstract

The tumor suppressor gene neurofibromin 1 (*NF1*) is a major regulator of the RAS‐MAPK pathway. *NF1* mutations occur in lung cancer but were not extensively explored. We hypothesized that *NF1*‐mutated tumors could define a specific population with a distinct clinical and molecular profile. We performed *NF1* sequencing using next generation sequencing (NGS) in 154 lung adenocarcinoma surgical specimens with known *KRAS*, *EGFR*, *TP53*, *BRAF*, *HER2*, and *PIK3CA* status, to evaluate the molecular and clinical specificities of *NF1*‐mutated lung cancers. Clinical data were retrospectively collected, and their associations with molecular profiles assessed. In this series, 24 tumors were *NF1* mutated (17.5%) and 11 were *NF1* deleted (8%). There was no mutation hotspot. *NF1* mutations were rarely associated with other RAS‐MAPK pathway mutations. Most of patients with *NF1* alterations were males (74.3%) and smokers (74.3%). Overall survival and disease‐free survival were statistically better in patients with *NF1* alterations (N = 34) than in patients with *KRAS *mutations (N = 30) in univariate analysis. Our results confirm that *NF1* is frequently mutated and represents a distinct molecular and clinical subtype of lung adenocarcinoma.

## 1 INTRODUCTION

Neurofibromin 1 (*NF1*) is a major tumor suppressor gene located on chromosome 17q11.2. *NF1* encodes a RAS (rat sarcoma)‐GAP (GTPase activating protein) known as neurofibromin. Neurofibromin facilitates the transit of RAS to their inactive state and functions as an inhibitor of the RAS‐mitogen‐activated protein kinase (MAPK) pathway.^1^ The RAS‐MAPK pathway has major implications in cancer biology, and drives cell differentiation, proliferation, and survival.^2^

Germline dominant loss‐of‐function mutations of the *NF1* gene cause the common inherited tumor predisposition syndrome neurofibromatosis type 1 (NF1; Online Mendelian Inheritance in Man database 162200). Tumor genome sequencing has resulted in the identification of somatic *NF1* mutations in various non‐NF1–associated sporadic cancers, including melanoma,^3^, ^4^ lung cancer,^5^ glioblastoma,^6^ ovarian cancer,^7^ breast cancer, and acute myeloid leukemia.^8^ More than 1500 mutations in the *NF1* gene have been reported in the *Human Gene Mutation Database* (HGMD), most of which are obvious loss­of­function alleles. The identification of *NF1* mutations remains challenging owing to the large size and structure of the gene, the presence of numerous pseudogenes, and the different types of mutations that can occur. Moreover, it is not yet known if biallelic or monoallelic loss of *NF1* contributes to tumor progression in sporadic cancers. Preclinical and clinical data suggested that treatment with MAP2K (MEK) inhibitor or in combination with mTOR inhibitors could be efficient to treat NF1‐associated tumors.^9^, ^10^

Lung adenocarcinoma is the most common form of lung cancer and has an average 5‐year survival rate of 15%, mainly because of late‐stage detection and a paucity of late‐stage treatments. Somatic activating mutations in the RAS‐MAPK pathway genes *KRAS, EGFR*, and *BRAF* were, respectively, identified in 30%, 14%, and 4% of lung adenocarcinoma with mutual exclusion in Caucasian population.^5^ Targeted therapies have been developed alone or in combination, allowing an increased in survival in patients with metastatic lung adenocarcinomas, especially in case of *EGFR* mutations. Adenocarcinomas in never‐smokers frequently contain mutations within the *EGFR* tyrosine kinase domain; those patients who often respond to tyrosine kinase inhibitor drugs (TKIs) usually develop drug resistance. Conversely, *KRAS* mutations are more common in ever‐smokers (former and current) and are associated with resistance to EGFR‐TKIs. Drug combinations including MEK inhibitors are currently under evaluation for *KRAS*‐mutated non–small‐cell lung carcinoma.^11^, ^12^

Although *NF1* is a major regulator of RAS‐MAPK pathway, only few clinical studies have described the pattern of *NF1* somatic mutations in lung adenocarcinoma.^5^, ^13^, ^14^ Using a targeted next generation sequencing (NGS) approach, we analyzed a large cohort of resected lung adenocarcinomas to characterize *NF1* mutations, and we evaluated the molecular and clinical specificities of *NF1*‐mutated lung cancers.

## 2 MATERIALS AND METHODS

### 2.1 Patients and samples

A total of 154 frozen tumor samples of primary lung adenocarcinoma were analyzed from 154 patients who underwent surgery between January 2001 and December 2006 in the Department of Thoracic Surgery of European Georges Pompidou Hospital (AP‐HP, Paris, France). Clinical and survival data were retrospectively collected. Patient age, gender, date of surgery, stage at diagnosis and at surgery (pTNM), history of smoking, treatment before surgery, date of relapse, and date of death were collected. Overall survival (OS) was calculated from the date of surgery to the date of death or last follow‐up. Disease‐free survival (DFS) was calculated from the date of surgery to the date of relapse or last follow up.

Samples were collected with appropriate consents that were reviewed and approved by regulatory and ethical authorities (CCP Île‐de‐France II n°2008‐136). Experienced pathologists histologically confirmed all cases, according to the 2004 World Health Organization (WHO) classification of lung neoplasms. Tumor and adjacent lung parenchyma were snap frozen in liquid nitrogen at the time of surgery and stored at −80°C. The Qiamp extraction kit (Qiagen) was used for subsequent extraction after proteinase K digestion. Most samples were previously analyzed for the following genes using Sanger sequencing as previously described by Blons et al^15^: *EGFR* (exons 18‐21), *TP53* (exons 4‐10), *KRAS* (exon 2), *BRAF* (exons 11 and 15), *HER2* (exons 18‐23), *PIK3CA* (exons 10 and 21), and *STK11* (exons 1‐9).^15^

### 2.2 NF1 sequencing

The coding sequence of the *NF1* gene was analyzed using a targeted NGS approach, as previously described.^16^ Experiments were performed on the NGS platform of the Cochin Hospital (AP‐HP, Paris, France). The targeted region included the entire *NF1*‐coding exons, intron boundaries (25 bp), and the 5′ and 3′ untranslated regions (UTRs). Next generation sequencing library preparation used the Ion AmpliSeq Library Kit 2.0 according to the manufacturer's instructions. The template‐positive ion sphere particles were loaded on Ion 318 chips and sequenced with an Ion PGM sequencer (Thermo Fisher Scientific).

Sequence alignment and extraction of SNPs and short insertions/deletions (indels) were performed using the Variant Caller plugin on the Ion Torrent Browser and DNA sequences visualized using the Integrated Genomics Viewer (IGV, v2.3) from Broad Institute. Major calling parameters were as follows: minimum allele frequency (MAF) ≥ 5% and minimum sequencing depth ≥200X for both SNPs and short indels. Variant annotation was performed using the PolyPhen‐2,^17^ Mutation Taster 18 and SIFT 19 software that provide in silico prediction of impact of an amino acid substitution. The creation of a new splice site was evaluated using the Human Splicing Finder V.2.4.1 (HSF) software.^20^ The assessment of variants implication was performed based on population databases (dbSNP and ExAC),^21^ mutation databases (*HGMD*), and predictions software. The criteria used for classifying missense variants as pathogenic were as follows: (a) MAF ≤ 0.1% in population databases, (b) in silico prediction with a “possibly damaging” or a “probably damaging” impact of the non‐synonymous variant on the structure and function of the protein, (c) in silico prediction of splicing alteration, and (d) report of the mutation by other groups or in mutations databases, as previously described.^22^

Identification of copy number alterations (CNAs) was performed using sequencing depth analysis. Read numbers for each separated *NF1* amplicon were normalized by dividing each amplicon read numbers by the total of amplicon read numbers of the control gene (*SPRED1*) from the same sample, as previously described.^16^ Normalized read numbers obtained for each amplicon of a sample were then divided by the average normalized read numbers of control samples for the corresponding amplicon. Copy number ratios of <0.7 and >1.3 were considered deleted and duplicated, respectively.

### 2.3 Statistical analysis

Associations between categorical variables were analyzed by a chi‐square test and between continuous variables by a Wilcoxon signed‐rank or *t* test. Survival was assessed by the Kaplan‐Meier method, and differences were analyzed by the log‐rank test. Variables significantly associated with OS (*P*‐value <0.05) on univariate analysis were included in multivariate analysis using a Cox proportional hazard regression model.

### 2.4 Ethics

The research was conducted according to the recommendations outlined in the Helsinki declaration. This study was approved by the institutional review board (CPP Ile de France II, 2008‐136).

## 3 RESULTS

### 3.1 NF1 sequencing

For a typical run of 24 samples, ~468 megabases (Mb) were generated, corresponding to 3.5 × 10^6^ reads. The mean read length was 141 bp. On average, for every sample, 99% of high quality sequencing reads (98% of bases) were mapped to the reference genome. This resulted in an evenly distributed mean sequencing depth for *NF1* of 656X. A good uniformity between samples and between amplicons was obtained. *NF1* was sequenced in 154 samples: of these, 17 samples were excluded from the subsequent analysis because the mean depth was <200X.

**Figure 1:**
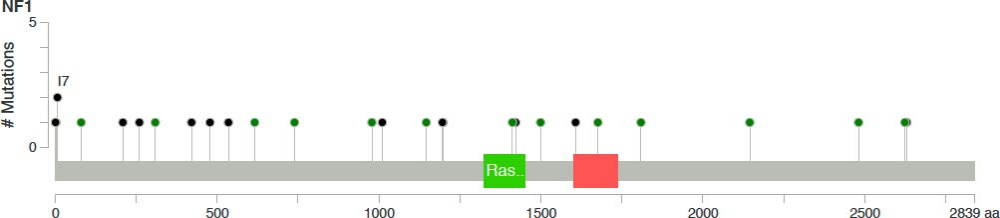
Neurofibromin 1 (*NF1*) point mutations distribution in the 24 of the 137 samples. Lollipop plot showed no hotspot mutation, using Mutation Mapper (cBioPortal). Green spots, missense mutations; black spots, truncating mutations; green rectangle, neurofibromin RasGAP domain; red rectangle, neurofibromin CRAL‐TRIO domain

**Figure 2:**
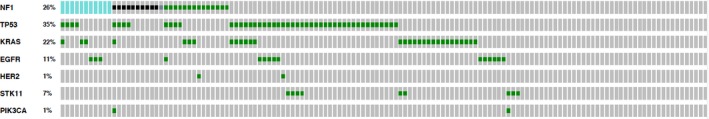
Oncoprint output of the genetic alterations in the 137 samples (green square, missense mutation; black square, nonsense and splice mutation; blue rectangle, deletion; gray rectangle, unaltered). The Oncoprint was obtained using OncoPrinter (cBioPortal)

Pathogenic *NF1* point mutations were identified in 24 of 137 (17.5%) samples: 6 non‐sense mutations, 13 missense mutations, 5 frameshift mutations, and 4 splice site mutations. Among them, 4 (16%) were compound heterozygous mutations. There was no mutation hotspot (Figure 1). The variant allele frequency (VAF) ranged from 5% to 91% and 3 of the 24 (8%) mutations had a VAF > 55% suggesting loss of heterozygosity (Table S1). In 11 of the 137 (8%) samples, *NF1* deletion was suggested by unbalanced copy number ratios using sequencing depth analysis (Figure 2).

### 3.2 Correlation with molecular characteristics

Among the 137 analyzed samples: *EGFR*, *KRAS,* and *PIK3CA* mutations were, respectively, found in 15 (10.9%), 30 (21.9%), and 2 (1.5%) samples (Figure 2). No *BRAF* mutation was found in the 86 tested samples. In addition, mutations were found in *TP53*: 48/136 (35.3%); *STK11*: 9/135 (6.7%); and *HER2:* 2/122 (1.6%). Samples with *NF1 *mutations samples were exclusive of *KRAS* or *EGFR* in 19 of the 24 cases (79%). Among the 24 samples with *NF1* point mutations, co‐mutations were found in *TP53:* 8 (33.3%); *KRAS*: 4 (16.7%); *EGFR*: 1 (4.2%); *PIK3CA:* 1 (4.2%); and *HER2:* 1 (4.2%; Figure 2, Tables S1‐S3). There was no *NF1* and *STK11* co‐mutation. Among the 11 samples with *NF1* deletions, co‐mutations were found in *EGFR* 3 (27.3%); *KRAS:* 3 (27.3%); and *TP53:* 4 (36.4%). No mutation in *STK11, PIK3CA,* or *HER2* was found in *NF1*‐deleted samples. Among the four samples with biallelic *NF1* alterations, only one had a *KRAS* co‐mutation (Tables S1, S2, and S4). *NF1* mutations and deletions were not statistically associated with *KRAS* mutations (*P* = 0.44 and *P* = 0.64, respectively) or *EGFR* mutations (*P* = 0.22 and *P* = 0.06, respectively).

### 3.3 Correlation with clinical characteristics

The cohort was mainly constituted of early lung adenocarcinoma stages: 70.1% of patients were with stage I or II disease (Table 1). Patients with *NF1 *alterations were more frequently men (74.3% vs 62% in the whole cohort), with significant enrichment in the *NF1* mutations subgroup (*P* = 0.04; Table S5). Past or current smoking status was reported for 104/137 (75.9%) patients in the whole cohort, 25/30 (83.3%) in the *KRAS* mutations subgroup, 39/44 (89.1%) in the *TP53* mutations subgroup, and 26/35 (74.3%) in the *NF1* alterations subgroup. There were less current or former smokers in the *EGFR* mutations subgroup (*P* < 0.0001; Table 2). The proportion of patients with early stage disease (stages I and II) was not different between the genotype subgroups: 97/137 (70.1%) in the whole cohort, 21/30 (70%) in the *KRAS*‐mutated group, 8/15 (53.3%) in the *EGFR*‐mutated group, 31/48 (64.6%) in the *TP53*‐mutated group, and 27/35 (77.1%) in the *NF1* alterations group. The proportion of patients with *NF1* alterations was not statistically different according to disease stage (*P* = 0.29): among patients with early stage disease (N = 96), 28.8% (27/96) had *NF1* alterations; among patients with advanced stage disease (N = 41), 19.5% (8/41) had *NF1* alterations (Table 2).

**Table 1:** Clinical characteristics of the cohort

Gender	
Female	52 (38%)
Male	85 (62%)
Mean age (y)	61.4 (32.8‐85.2)
Tobacco	
Yes	104 (75.9%)
No	21 (15.3%)
Unknown	12 (8.8%)
Stage^a^	
I	73 (53.3%)
II	23 (16.8%)
III	28 (20.4%)
IV	13 (9.5%)
Chemotherapy or radiochemotherapy before surgery	
Yes	9 (6.6%)
No	128 (93.4%)

a For each patient, the stage was established thanks to surgical samples. Nine patients received chemotherapy or chemoradiotherapy before surgery. For these nine samples, the stage was established after receiving systemic treatments. Pathological stage is detailed in the Table S2.

**Table 2:** Clinical characteristics according to molecular profile

	All population (N = 137)	*NF1* alterations (N = 35)	*P* *	*KRAS* mutations (N = 30)	*P* **	*EGFR* mutations (N = 15)	*P* ***
Gender							
Female	52 (38%)	9 (25.7%)	0.12	12 (40%)	0.83	9 (60%)	0.07
Male	85 (62%)	26 (74.3%)		18 (60%)		6 (40%)	
Mean age	61.4 (32.8‐85.2)	61.3 (46.1‐76.4)	0.92	59.7 (41.9‐81.7)	0.32	63.2 (50.3‐84)	0.48
Tobacco							
Yes	104 (75.9%)	26 (74.3%)		25 (83.3%)		5 (33.3%)	
No	21 (15.3%)	6 (17.1%)	0.79	2 (6.7%)	0.15	7 (46.7%)	**<0.0001**
Unknown	12 (8.8%)	3 (8.6%)		3 (10%)		3 (20%)	
Stage							
I‐II	96 (70.1%)	27 (77.1%)	0.29	21 (70%)	0.99	8 (53.3%)	0.13
III‐IV	41 (29.9%)	8 (22.9%)		9 (30%)		7 (46.7%)	
Chemotherapy or radiochemotherapy before surgery							
Yes	9 (6.6%)	5 (14.3%)	**0.04**	3 (10%)	0.53	1 (6.7%)	0.91
No	128 (93.4%)	30 (85.7%)		27 (90%)		14 (93.3%)	

* Statistical analysis between patients with *NF1* alterations (N = 35) and patients without *NF1* alteration (N = 102).

** Statistical analysis between patients with *KRAS* mutations (N = 30) and patients without *KRAS* mutation (N = 107).

*** Statistical analysis between patients with *EGFR* mutations (N = 15) and patients without *EGFR* mutation (N = 122).

Bold values mean that the result is statistically significant.

### 3.4 Univariate analyses for DFS and OS according to molecular subgroups

**Figure 3:**
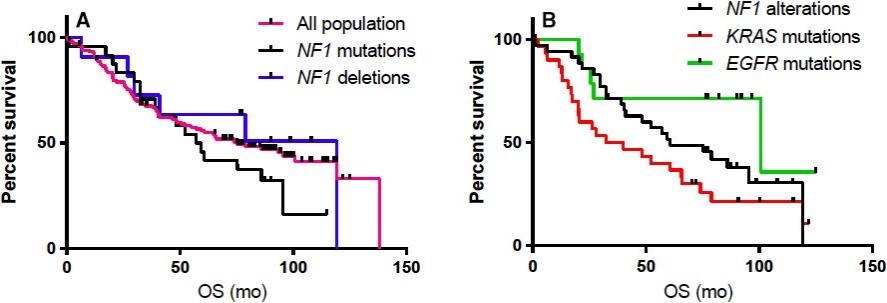
A, Kaplan‐Meier curve comparing overall survival (OS) of patients with neurofibromin 1 (*NF1*) point mutations (N = 24, black curve), *NF1* deletions (N = 11, blue curve), and without *NF1* alterations (N = 102, pink curve). No statistical difference was found (log‐rank test: *P* = 0.5). B, Kaplan‐Meier curve comparing OS of patients with *NF1* alterations (N = 35, black curve), *KRAS* mutations (N = 30, red curve), and *EGFR* mutations (N = 15, green curve). Patients with *NF1* alterations have a significantly higher survival than patients with *KRAS* mutations (log‐rank test: *P* < 0.0001)

No statistical difference in DFS and OS was found between patients with *NF1* alterations (point mutation or deletions) or without *NF1* alterations (Figure 3A). Disease‐free survival was 57.1 months in patients without *NF1* alteration vs 43.1 months in the *NF1* alterations group (*P* = 0.3), 92.6 months in the *EGFR* group (*P* = 0.5), and 25.7 months in the *KRAS* mutations group (*P* = 0.01). Disease‐free survival in the *NF1* alterations group was statistically better than in the *KRAS* mutations group (*P* = 0.004).

Overall survival was 60.7 months in the *NF1* alterations group, 36.1 months in the *KRAS* mutations group, 100.6 months in the *EGFR* mutations group, and 75.2 months in the entire cohort (Figure 3B). There was no statistical OS difference between patients with or without *NF1* point mutations (*P* = 0.42), and between patients with or without *NF1* deletion (*P* = 0.52). There was a trend for an increased OS in *NF1*‐deleted vs *NF1*‐mutated patients: 119.1 vs 58.2 months, respectively (*P* = 0.06). Overall survival was significantly shorter in patients with *KRAS *mutations vs patients without *KRAS* mutations (*P* = 0.009) and vs patients with *NF1* mutations (*P* = 0.004). The median OS in *NF1*‐ and *KRAS*‐co‐mutated patients (52.1 months) was significantly decreased vs *NF1*‐mutated patients without *KRAS* mutations (81 months; *P* = 0.03). No statistically significant difference was found in *KRAS*‐mutated patients without *NF1* mutations (27.9 months; *P* = 0.59).

### 3.5 Multivariate analysis

In multivariate analysis (including age, tumor stage, *KRAS* mutations, and *NF1* alterations), *KRAS* mutations were strongly independently associated with a poor prognosis (*P* = 0.004, Hazard ratio, HR = 2.5), as well as *KRAS* and *NF1* co‐mutations (*P* = 0.04, HR = 2.3) and advanced tumor stage (*P* < 0.0001, HR = 2.9; Table 3). *NF1*‐altered/*KRAS* wild‐type (WT) patients did not show a worse OS compared with *NF1* WT/*KRAS* WT patients.

**Table 3:** Multivariate Cox Model on overall survival

Characteristics	Hazard ratio	95% CI	*P*
Age (per year)	1.01	0.98‐1.03	0.58
Tumor stage (I‐II vs III‐IV)			
I‐II (ref)	1	–	**–**
III‐IV	2.91	1.79‐4.71	**<0.0001**
Mutations			
*KRAS* WT/*NF1* WT (ref)	1	–	**–**
*KRAS* mutations/*NF1* WT	2.47	1.33‐4.59	**0.004**
*NF1* alterations/*KRAS* WT	1.44	0.78‐2.67	0.24
*NF1* and *KRAS* co‐mutations	3.35	1.03‐5.36	**0.04**

ref, reference; WT, Wild type.

Bold values mean that the result is statistically significant.

In multivariate analyses separating *NF1* mutations and *NF1* deletions, *NF1* mutations or deletions were not associated with prognosis. *KRAS* mutations were independently associated with poor prognosis regardless of *NF1* alteration status.

### 3.6 Impact of neoadjuvant treatment

In the whole cohort, only 9 of the 137 (6.6%) patients received neoadjuvant chemotherapy or radiochemotherapy. Among them, 5 (of the 9) had *NF1* alterations, including 4 heterozygous point mutations (1 non‐sense, 2 missense, and 1 splice site mutation), and one large deletion. Patients with *NF1* were significantly more often treated with chemotherapy: 6.6% (5/35) of patients without *NF1* alterations received chemotherapy while 14.3% (9/137) of patients with *NF1* mutations received chemotherapy before surgery (*P* = 0.01). The median age of these five *NF1* patients was 55.1 years (46.1‐69.5). Of the five patients, three were men and three were current or former smokers (Tables S1 and S2). In two of the five *NF1*‐mutated tumors, paired pre‐ and post‐chemotherapy samples were analyzed. The same *NF1* mutation was identified in both pre‐ and post‐chemotherapy paired samples.

## 4 DISCUSSION

In our series, *NF1* mutations and deletions were found in 17.5% and 8% of lung adenocarcinoma surgical specimens, respectively. Among the 24 *NF1* mutations, 4 (16.7%) were homozygous. Most *NF1* mutations were exclusive of *KRAS* and *EGFR* mutation (19 of the 24 samples)*,* and one third co‐occurred with *TP53*. These results are consistent with previous published studies.^14^, ^23^, ^24^ However, the occurrence of *NF1* mutations was higher in our study (17.5%) compared to the study by Redig et al (10%).^14^ The population of these two studies was different: Redig et al study described patients with metastatic adenocarcinomas and squamous cell cancers and our cohort only included patients with metastatic adenocarcinoma. This difference may explain the higher occurrence of *NF1* mutations in our cohort. Moreover, the method used for variant selection was different between the two studies. Redig et al^24^ used a MAF ≥ 10% and a minimum sequencing depth ≥50X for variant detection. In the present study, calling parameters were a MAF ≥ 5% and a minimum sequencing depth ≥200X. In our study, four *NF1*‐mutated samples have a MAF comprised between 5% and 10%. If we exclude these samples, *NF1* mutations are found in 20 of the 137 samples (14.6%) in our cohort (Table S1).

Co‐occurrence of *NF1* alterations with *EGFR* and *KRAS* mutations were rare in our series, suggesting a driver role. Even in cases of co‐mutations in RAS‐MAPK pathway genes, *NF1* alterations were predicted to be deleterious (Table S1).

The clinical profile of *NF1*‐mutated patients was similar to the one of *KRAS*‐mutated patients who were mainly males, and current or former smokers. Previous studies demonstrated that *KRAS* mutation had a negative prognostic impact, especially in early stage lung cancer.^26^, ^27^ Here, we observed that *NF1*‐mutated patients showed significantly increased DFS and OS vs *KRAS*‐mutated patients in univariate analysis (Figure 3). In multivariate analyses for OS, *KRAS* mutations were found to be associated with poorer survival both in *NF1* WT and *NF1*‐altered status. *NF1*‐altered patients with no *KRAS* mutation had the same prognosis than *NF1* WT/*KRAS* WT patients. Few data are available concerning *NF1*‐mutated patients' prognosis. Redig et al and Pan et al did not find differences in survival between *NF1*‐ and *KRAS*‐mutated patients.^14^, ^23^ However, the studied populations were different than in the present study. Our results have to be confirmed in a larger cohort.

The clinical and molecular profiles of *NF1*‐deleted (11/137) and *NF1*‐mutated (24/137) patients were different. Patients with *NF1* deletions included less smokers, younger patients, more females, and showed a trend for a better survival compared to patients with *NF1* point mutation (Table S5). To our knowledge, no previous study had reported this observation. In our cohort, three *NF1* deletions (27.3%) were associated with *EGFR* mutations. Only few previous data described *NF1* CNAs in lung cancers and no co‐occurrence of *NF1* deletions and *EGFR* mutations was found.^23^, ^28^, ^29^ The co‐occurrence of *NF1* deletions with *EGFR* mutations could explain the observed associated phenotype in our cohort.

Only surgery specimen samples were analyzed in our cohort explaining the small proportion of stage IV disease (9.5%) and the small proportion of patients receiving chemotherapy (6.6%) at the time of the surgery. However, we report no significant difference in disease stages according to *NF1* molecular status maybe because of the small cohort size. Interestingly, we found a significant higher proportion of *NF1* mutations in tumors from chemotherapy‐treated patients vs treatment‐naive patients. Even if emergence of *NF1* mutations following targeted therapies was previously described in melanoma, and the downregulation of *NF1* expression was observed in *EGFR*‐mutated lung adenocarcinoma,^31^, ^32^ we do not confirm this observation in our cohort. The same *NF1* mutations were identified in the two available pre‐ and post‐chemotherapy paired samples. Our observation of a significant higher proportion of *NF1* mutations in tumors from chemotherapy‐treated patients needs to be confirmed in a larger cohort.

We identified 4 of the 24 (16.7%) *NF1* compound heterozygous mutations. The four patients with biallelic *NF1* mutations had the same clinical characteristics than patients with monoallelic *NF1* alterations. Only one patient had a co‐mutation with *KRAS* (Tables S1‐S3). In a previous study, Redig et al also reported 15% (9/60) of lung adenocarcinoma samples with biallelic *NF1* mutations. Little is known on functional consequences of *NF1* mutation allelic balance in cancer cells.^33^, ^34^, ^35^ It has become apparent that not all consistent loss‐of‐function hits in tumor suppressor genes are accompanied by obvious aberrations on the WT allele, in discordance with the two‐hit hypothesis affecting tumor suppressor genes.^36^ Single‐copy loss may have a role in tumorigenesis, and haploinsufficiency effect may be highly tissue specific and context dependent. An appreciation of the functional role of *NF1* gene dosage in lung adenocarcinoma development will be important for prediction of response to therapeutics. Notably, preclinical and clinical data have suggested efficacy of targeted therapies including MEK or mTOR inhibitors alone or in combination in *NF1*‐mutated tumors.^10^, ^37^, ^38^ It will be important not only to assess the presence of *NF1* mutation in a tumor, but also to accurately assess the potential therapeutic impact of *NF1* point mutations, copy number, and the ratio of mutant to normal as predictive biomarkers.^39^

## 5 CONCLUSION

Our results are consistent with previous published data and confirm the implication of *NF1* somatic alterations in lung adenocarcinoma with distinct molecular and clinical characteristics. These findings need to be confirmed in a larger cohort and functional consequences to be studied for a better management of available treatments including chemotherapy, targeted therapies, or immunotherapies.

## CONFLICT OF INTEREST

The authors have no conflict of interest.
