# Supplementary material for: NF1 mutations identify molecular and clinical subtypes of lung adenocarcinomas
Source: Cancer Med. 2019 Jun 14;8(9):4330–7. doi: 10.1002/cam4.2175 (PMC6675708; doi:10.1002/cam4.2175)
Supplement: Supplementary file 1 [file CAM4-8-4330-s001.pdf]

Supplementary Table 1: Molecular characteristics of *NF1* point mutations.

Patients	Exon/Intron (NCBI)	c.DNA NM_000267.3	Protein	VAF* (%)	Type of mutation	SIFT	Mutation Taster	Polyphen	HSF	ExAC MAF** (%)	Co-mutations
75	Exon 22	c.2932G>T	p.Gly978Cys	18	Missense	0.01 Deleterious	1 Disease causing	1 Probably damaging	-	-	*TP53*
85	Exon 17	c.1848G>T	p.Gln616His	13	Missense	0.13 Tolerated	0.973 Disease causing	0.914 Probably damaging	-	-	*TP53*
109	Intron 1	c.60+7G>C	p.(?)	68	Splice	-	-		Probable effect	-	*KRAS*
115	Exon 27	c.3588dup	p.Ala1197Cysfs*7	18	Nonsense	-	-	-	-	-	*TP53*<br>*KRAS*<br>*PIK3CA*
	Exon 13	c.1432A>T	p.Lys478*	22	Nonsense	-	-	-	-	-	
128	Exon 43	c.6371C>A	p.Thr2124Asn	28	Nonsense	0.3 Tolerated	1 Disease causing	0.889 Probably damaging	-	-	-
132	Exon 17	c.1849del	p.Ala617Glnfs*14	91	Nonsense	-	-		-	-	-
137	Exon 50	c.7378G>T	p.Gly2460Cys	6	Missense	0.1 Tolerated	0.656 Disease causing	0.115 Benign	-	-	-
160	Exon 37	c.4963G>A	p.Ala1655Thr	18	Missense	0.3 Deleterious	1 Disease causing	0.943 Probably damaging	-	0.00082	*TP53*
161	Intron 29	c.3975-2A>T	p.(?)	11	Splice	-	-	-	Probable effect	-	*TP53*
	Exon 36	c.4760del	p.Leu1587*	16	Nonsense	-	-	-	-	-	-
167	Exon 6	c.625C>T	p.Gln209*	10	Nonsense	-	-	-	-	-	-
177	Exon 34	c.4432A>G	p.Ile1478Val	15	Missense	0.85 Tolerated	1 Disease causing	0.950 Probably damaging	-	0.0016	*KRAS*

178	Exon 32	c.4172G>T	p.Arg1391Ile	48	Missense	0 Deleterious	1 Disease causing	0.994 Probably damaging	-	-	-
181	Exon 9	c.925G>T	p.Gly309Cys	11	Missense	0.02 Deleterious	1 Disease causing	0.997 Probably damaging	-	-	-
182	Exon 12	c.1263C>T	p.Ser421Ser	49	Splice	-	-	-	Probable effect	0.0016	-
187	Exon 54	c.7827_7828insCA	p.Thr2610Glnfs*14	59	Nonsense	-	-	-	-	-	*TP53*
222	Exon 32	c.4203T>G	p.Tyr1401*	24	Nonsense	-	-	-	-	-	-
	Intron 49	c.7259-1G>T	p.(?)	27	Splice	-	-	-	Probable effect	-	
224	Exon 26	c.3436G>A	p.Val1146Ile	48	Missense	0.89 Tolerated	1 Disease causing	0.902 Probably damaging	-	-	-
227	Exon 8	c.780del	p.Lys261Asnfs*20	19	Nonsense	-	-	-	-	-	-
	Exon 54	c.7870G>A	p.Ala2624Thr	20	Missense	0.45 Tolerated	1 Disease causing	0.718 Probably damaging	-	-	
228	Exon 3	c.238T>C	p.Tyr80His	22	Missense	0.02 Deleterious	1 Disease causing	0.997 Probably damaging	-	-	*KRAS*
230	Exon 38	c.5363C>T	p.Pro1788Leu	19	Missense	0.16 Tolerated	1 Disease causing	1 Probably damaging	-	0.0049	*EGFR TP53*
240	Exon 23	c.3028C>T	p.Gln1010*	20	Nonsense	-	-	-	-	-	-
249	Exon 27	c.3592G>T	p.Glu1198*	5	Nonsense	-	-	-	-	-	-
259	Exon 14	c.1603C>T	p.Gln535*	5	Nonsense	-	-	-	-	-	*TP53*
263	Exon 18	c.2215A>G	p.Met739Val	6	Missence	0.48 Tolerated	0.998 Disease causing	0.002 Benign	-	-	*HER2*

*VAF: Variation Allele Frequency; **MAF: Minor Allele Frequency

Supplementary Table 2: Clinical characteristics of patients with *NF1* alterations.

Patients	*NF1* alterations (NM_000267.3)	Co-mutations	Gender	Age (years)	Tobacco	Stage	Chemotherapy or radio chemotherapy before surgery
75	c.2932G>T	*TP53*	Male	58.8	Yes	pT3N2M0	No
85	c.1848G>T	*TP53*	Male	56.5	Unknown	ypT1bN0M0*	Yes
109	c.60+7G>C	*KRAS*	Male	51.7	Yes	ypT2aN0M0*	Yes
115	c.3588dup<br>c.1432A>T	*TP53*<br>*KRAS*<br>*PIK3CA*	Female	58.2	Yes	pT1N0M0	No
128	c.6371C>A	-	Male	60.3	Yes	pT1aN0M0	No
132	c.1849del	-	Male	59.2	Unknown	pT2N0M0	No
137	c.7378G>T	-	Male	58.6	Yes	pT2aN2M0	No
160	c.4963G>A	*TP53*	Male	71.7	Yes	pT2aN0M0	No
161	c.3975-2A>T<br>c.4760del	*TP53*	Male	73.6	Yes	pT2aN0M0	No
167	c.625C>T	-	Male	73.1	Yes	pT1aN0M0	No
177	c.4432A>G	*KRAS*	Male	53.7	Yes	pT1aN0M0	No
178	c.4172G>T	-	Female	55.4	Yes	pT3N0M0	No
181	c.925G>T	-	Male	69.5	Yes	pT3N0M0	No
182	c.1263C>T	-	Female	66.1	Unknown	pT2aN0M0	No
187	c.7827_7828insCA	*TP53*	Male	54.7	Yes	pT1aN0M0	No
222	c.4203T>G<br>c.7259-1G>T	-	Female	56.1	Yes	pT2aN0M0	No
224	c.3436G>A	-	Female	69.5	No	ypT2aN2M0*	Yes
227	c.780del<br>c.7870G>A	-	Male	46.5	No	pT2aN0M0	No
228	c.238T>C	*KRAS*	Male	63.2	Yes	pT3N0M0	No
230	c.5363C>T	*EGFR*	Male	58.9	Yes	pT3N0M1a	No

		TP53					
240	c.3028C>T	-	Male	69.2	Yes	pT1aN0M0	No
249	c.3592G>T	-	Male	46.1	Yes	ypT2aN0M0*	Yes
259	c.1603C>T	*TP53*	Male	68.5	Yes	pT2aN0M0	No
263	c.2215A>G	*HER2*	Male	76.4	Yes	pT1aN0M0	No
15	Deletion	*KRAS*	Female	72.6	Yes	pT2aN0M0	No
44	Deletion	*TP53*	Male	62.5	Yes	pT3N0M0	No
106	Deletion	*TP53*	Male	55.9	Yes	pT1aN0M0	No
124	Deletion	*TP53*	Male	76.1	Yes	pT2bN0M0	No
141	Deletion	*KRAS*	Male	76.3	Yes	pT3N0M1b	No
170	Deletion	*EGFR*	Male	65.4	No	pT2aN0M0	No
171	Deletion	-	Female	43.3	Yes	pT3N0M0	No
229	Deletion	*TP53* *KRAS*	Female	74.3	No	pT4N2M1b	No
233	Deletion	-	Male	70.6	Yes	pT2aN0M0	No
244	Deletion	*EGFR*	Female	58.9	No	pT1aN0M0	No
288	Deletion	*EGFR*	Female	52	No	ypT3N0M1b *	Yes

***** pathologic TNM stage assessed in surgical specimens after chemotherapy

Supplementary Table 3: Clinical and molecular characteristics of *NF1* and *KRAS* mutated patients.

	NF1 alterations (NM_000267.3)	Co-mutations	Gender	Age (years)	Tobacco	Stage	Chemotherapy pre-treated*
**Patient 109**	c.60+7G>C, Splice site mutation	No	Male	51.7	Yes	ypT2aN0M0	Yes
**Patient 115**	c.3588dup, p.Ala1197Cysfs*<br>c.1432A>T, p.Lys478*	*TP53*<br>*PIK3CA*	Female	58.2	Yes	ypT1N0M0	No
**Patient 177**	c.4432A>G, p.Ile1478Val	No	Male	53.7	Yes	ypT1aN0M0	No
**Patient 228**	c.238T>C, pTyr80His	No	Male	63.2	Yes	ypT3 N0M0	No
**Patient 141**	Deletion	No	Male	71.3	Yes	ypT3N0M1b	No
**Patient 229**	Deletion	*TP53*	Female	74.3	No	ypT4N2M1b	No
**Patient 15**	Deletion	No	Female	72.6	Yes	ypT2aN0M0	No

*pathologic TNM stage assessed in surgical specimens after chemotherapy

Supplementary Table 4: Clinical and molecular characteristics of patients with bi-allelic *NF1* point mutations.

	***NF1* alterations (NM_000267.3)**	**Co-mutations**	**Gender**	**Age (years)**	**Tobacco**	**Stage**	**Chemotherapy pre-treated***
**Patient 227**	c.779delC, p.Thr260fs<br>c.7870G>A, p.Ala2624Thr	No	Male	46.7	No	ypT2aN0M0	No
**Patient 115**	c.1432A>T, p.Lys478*<br>c.3588dup, p.Ala1197Cysfs*	*KRAS*<br>*TP53*<br>*PIK3CA*	Female	58.2	Yes	ypT1N0M0	No
**Patient 161**	c.29-2A>T, Splice site mutation<br>c.4821delT, p.Tyr1607fs	*TP53*	Male	72.2	Yes	ypT2N0M0	No
**Patient 222**	c.49-1G>T, Splice site mutation<br>c.4266T>G, p.Tyr1422*	No	Female	56.1	Yes	ypT2aN0M0	No

*pathologic TNM stage assessed in surgical specimens after chemotherapy

Supplementary Table 5: Clinical characteristics according to *NF1* alterations

	All population (N=137)	*NF1* mutations (N=24)	p*	*NF1* deletions (N=11)	p**
**Gender:**					
• **Female**	52 (38%)	4 (16.7%)	**0.04**	5(45.4%)	0.61
• **Male**	85 (62%)	20 (83.3%)		6(54.6%)	
**Mean age (years)**	61.4 (32.8-85.2)	60 (46.1-76.4)	0.51	59.9 (52-76.1)	0.29
**Tobacco:**					
• **Yes**	104 (75.9%)	19 (79.2%)	0.09	7 (63.6%)	**0.04**
• **No**	21 (15.3%)	2 (8.3%)		4 (36.4%)	
• **Unknown**	12 (8.8%)	3 (12.5%)		0 (0%)	
**Stage:**					
• **I-II**	96 (70.1%)	19 (79.2%)	0.28	8 (72.7%)	0.29
• **III-IV**	41 (29.9%)	5 (20.8%)		3 (27.3%)	
**Chemotherapy or radio chemotherapy before surgery:**					
• **Yes**	9 (6.6%)	4 (16.7%)	**0.01**	1 (9.1%)	0.81
• **No**	128 (93.4%)	20 (83.3%)		10 (90.9%)	

* Statistical analysis between patients with *NF1* mutations (N=24) and patients without *NF1* mutation (N=113)

** Statistical analysis between patients with *NF1* deletions (N=11) and patients without *NF1* deletion (N=126)
